# Impact of Universal Screening for HDV in HBV‐Infected Patients on Chronic HDV Detection Rate in Israel

**DOI:** 10.1111/jvh.70046

**Published:** 2025-06-11

**Authors:** David Yardeni, Omer Cividalli, Bryan Itkowitz, Inna Lipnizkiy, Ali Abu Juma'a, Naim Abufreha, Ayelet Keren‐Naus, Nadav Eisner, Anat Nevo Shor, Ohad Etzion

**Affiliations:** ^1^ Department of Gastroenterology and Liver Diseases Soroka University Medical Center Beersheba Israel; ^2^ Joyce and Irving Goldman Medical School Faculty of Health Sciences Beersheba Israel; ^3^ Clinical Research Center Soroka University Medical Center Beersheba Israel; ^4^ Laboratory of Virology Services Soroka University Medical Center Beersheba Israel

**Keywords:** hepatitis B, hepatitis D, universal screening

## Abstract

Hepatitis delta virus (HDV) and hepatitis B virus (HBV) co‐infection is considered a progressive chronic viral hepatitis where treatment options are limited and significant morbidity and mortality are prevalent. Studies have shown insufficient testing for HDV antibody (anti‐HDV) among HBV‐infected patients. Unlike European and Asian‐Pacific guidelines, the American Association for the Study of Liver Diseases (AASLD) guidelines recommend HDV testing only for high‐risk HBV patients. We evaluated the efficacy of universal vs. risk‐based screening in identifying HDV infection among HBV patients. We performed a retrospective analysis of patients diagnosed with a positive HBsAg in a tertiary medical center and screened for HDV between 2010 and 2022. 761 patients were found to be HBsAg‐positive. 525 (69%) patients met AASLD criteria for HDV screening (high‐risk) and 236 (31%) did not (low‐risk). Universal screening was performed on 559 (73.4%) patients. In the high‐risk group, anti‐HDV positivity was found in 33 patients (8.6%). 17 (51.5%) were found to be HDV RNA‐positive. In the low‐risk group, 4 (2.3%) were found to be anti‐HDV‐positive. None were found to be HDV RNA‐positive. Screening based on AASLD criteria identified only 89% of HDV antibody‐positive patients. During the study period, an increased rate of all‐cause mortality was observed in the AASLD high‐risk group. In this single‐center study, universal screening of HBsAg‐positive patients identified 11% more anti‐HDV‐positive patients in comparison to the AASLD‐supported high‐risk‐only screening recommendations. Due to the paramount importance of HDV detection, universal HDV screening in HBsAg‐positive patients is encouraged.

**Abbreviations**
AASLD: American Association for the Study of Liver Diseases
AHR: AASLD high‐risk group
ALR: AASLD low‐risk group
ALT: alanine aminotransferase
AST: aspartate aminotransferase
EASL: European Association for the Study of the Liver
EMA: European medicine agency
FDA: Food and Drug Administration
HBV: hepatitis B virus
HCC: hepatocellular carcinoma
HDV: hepatitis delta virus
HIV: human immunodeficiency virus
HR: hazard ratio
NA: nucleos(t)ide analogues
SUMC: Soroka University Medical Center

## 1 Introduction

Hepatitis D virus (HDV) is an obligatory satellite of Hepatitis B virus (HBV), as it uses the helper virus envelope proteins (HBsAg) to create its viral particles [1]. HBsAg is embedded within the lipid viral envelope that protects the HDV nucleocapsid and allows the virus to penetrate other hepatocytes. Thus, an infection with HBV is indispensable for the virus to replicate within the host hepatocyte and generate virions. When afflicted with HDV, the patient may manifest the most severe form of human viral hepatitis known, frequently associated with the development of cirrhosis and a high likelihood of hepatocellular carcinoma (HCC) [2]. As of today, chronic HDV infection has no United States Food and Drug Administration (FDA)‐approved therapy and is considered incurable [3]. Recently, the European Medicines Agency (EMA) approved the entry inhibitor bulevirtide as a novel therapy; however, long‐term implications of this treatment are still pending [4].

Much debate exists regarding the current epidemiology of HDV. It is clear that a significant difference exists between the widespread HBV infection, which chronically infects nearly 300 million individuals worldwide [5, 6] and the much lower prevalence of HDV [7]. However, there are also significant differences in current estimations regarding the true worldwide prevalence of HDV [8, 9, 10, 11, 12]. According to the most recent studies, the number of estimated HDV patients ranges between 12 million to over 70 million individuals globally. Reasons for these wide variations in the estimation of global HDV prevalence include geographic differences and insufficient testing for HDV antibodies among HBV [13]‐monoinfected or human immunodeficiency virus (HIV)/HBV‐co‐infected patients [14]. Further complicating the determination of the epidemiological data is a lack of standardisation in the detection methods employed by various labs in the identification and quantification of HDV RNA [15, 16] often leading to false‐negative results.

Guidelines from the American Association for the Study of Liver Diseases (AASLD) recommend HDV screening for HIV‐positive persons, persons who inject drugs, men who have sex with men, those at risk for sexually transmitted diseases and immigrants from areas of high HDV endemicity [17]. Patients with low HBV‐DNA levels and elevated alanine aminotransferase (ALT) or aspartate aminotransferase (AST) levels may also be considered for HDV screening. On the other hand, HBV and HDV guidelines from the European Association for the Study of the Liver (EASL) and the Asian Pacific Association for the Study of the Liver simply promote universal anti‐HDV screening as part of the robust evaluation recommended for the HBV patient [18, 19, 20].

In Israel, no formal guidance has been issued regarding HDV screening in the HBsAg‐positive population, and therefore anti‐HDV testing is based on the index of suspicion of the treating physician. In agreement with EASL guidelines, since 2010, Soroka University Medical Center (SUMC), a large tertiary care hospital in Southern Israel, has adopted a local policy of universal screening for HDV in all newly diagnosed HBsAg‐positive patients. In this study, we set out to evaluate the sensitivity of universal vs. the AASLD risk‐based screening in identifying HDV infected patients among patients with chronic HBV infection.

## 2 Materials and Methods

### 2.1 Study Population

We retrospectively investigated male and female patients above the age of 1 year who were diagnosed with HBV via a new positive test for HBsAg at SUMC virology lab between January 2010 and September 2022. The SUMC virology lab provides services for a population of over 1 million people living in the Negev region of Israel. Patients lacking sufficient clinical data needed to establish criteria for AASLD [17] risk‐based HDV screening were excluded.

All patients were identified by a national ID number and were members of Clalit Health Services (CHS), the largest health maintenance organisation (HMO) in Israel. CHS maintains a computerised database, with complete records of patients' medical history, laboratory and imaging test results, medications and mortality data.

### 2.2 Data Sources and Clinical Definitions

Clinical parameters including demographics, diagnosis, laboratory and imaging data were collected if they were within 1 year of the new HBV diagnosis. Active hepatitis was identified when a patient presented with an ALT of above 56 U/L (twice above the SUMC lab upper limit of norm). Patients diagnosed with positive anti‐HDV were identified based on the LIAISON XL MUREX Anti‐HDV assay from DiaSorin, Saluggia, Italy. A positive HDV RNA PCR test was detected via an in‐house‐based Real‐Time Qualitative PCR assay operating on the Cobas TaqMan Platform [21]. Testing for HDV was performed only in patients where a second blood sample was provided following a new diagnosis of HBV.

Following acquisition of all retrospective data, we applied the AASLD guidelines to categorise the HDV patients into two study groups: the subpopulation of patients who would have been identified according to the AASLD guidelines (positivity of at least one of the AASLD guidelines parameters) and patients who were only identified through the implementation of universal screening. The following criteria were implemented: persons born in regions with reported high HDV endemicity (Table S1), persons who have ever injected drugs, men who have sex with men, individuals infected with HCV or HIV, persons with any history of sexually transmitted disease, and elevated ALT or AST with low or undetectable HBV DNA. The criteria regarding sexual practices was not implemented in our study due to lack of data on this criteria in the patient EMR. In addition, the criteria regarding individuals with elevated ALT or AST with low or undetectable HBV DNA was only utilised when HBV DNA data was available during the initial diagnosis and treatment with nucleos(t)ide analogues (NA) was yet to be initiated.

### 2.3 Study Outcomes

The primary hypothesis for the study was that due to the low rates of HDV positivity in our region, HDV screening in agreement with current AASLD guidelines will potentially miss a substantial number of patients while universal screening will show a greater yield in capturing patients co‐infected with HDV. The primary clinical outcome evaluated was HDV screening positivity via universal screening in comparison to HDV screening following AASLD guidelines. Secondary outcomes included HDV RNA positivity among patients identified via universal screening in comparison to patients identified per AASLD guidelines and clinical differences in the severity of liver disease and all‐cause mortality between patients proclaimed to be high risk for HDV by the AASLD criteria and patients considered low risk for HDV infection.

### 2.4 Ethics Statement

The study was approved by the institutional review board of SUMC and was conducted in compliance with the Declaration of Helsinki, Good Clinical Practice guidelines and local regulatory requirements. The Institutional Review Board of SUMC for studies on existing patient data decided for request number 0012‐23‐SOR on a full waiver of informed consent that was given on February 22, 2023, for study protocol SCRC22062.

### 2.5 Statistical Analysis

Patient characteristics were described using frequencies for categorical variables and means for continuous variables. When appropriate, univariate comparisons were made using χ2‐test or Fisher's exact test for categorical variables and using analysis of variance (t test) or Kruskal–Wallis tests for quantitative variables. The log‐rank test was employed to compare survival distributions between the groups. To explore the relationship between AASLD risk status and survival outcomes, a univariate Cox proportional hazards model was initially fitted. This was followed by a multivariable Cox regression model to adjust for potential confounders. Covariates were selected based on their clinical relevance, potential influence on survival and supporting evidence from prior literature. Results were reported as hazard ratios (HRs) with 95% CIs, quantifying the strength and direction of associations. A *p*‐value of 0.05 or less (two‐sided) was considered statistically significant. All analyses were conducted using R (version 2023.09.1 + 494). Key packages, including survival, survminer and gtsummary, facilitated survival analysis, visualisation and the generation of regression tables.

## 3 Results

### 3.1 Study Population and HDV Screening

**FIGURE 1:**
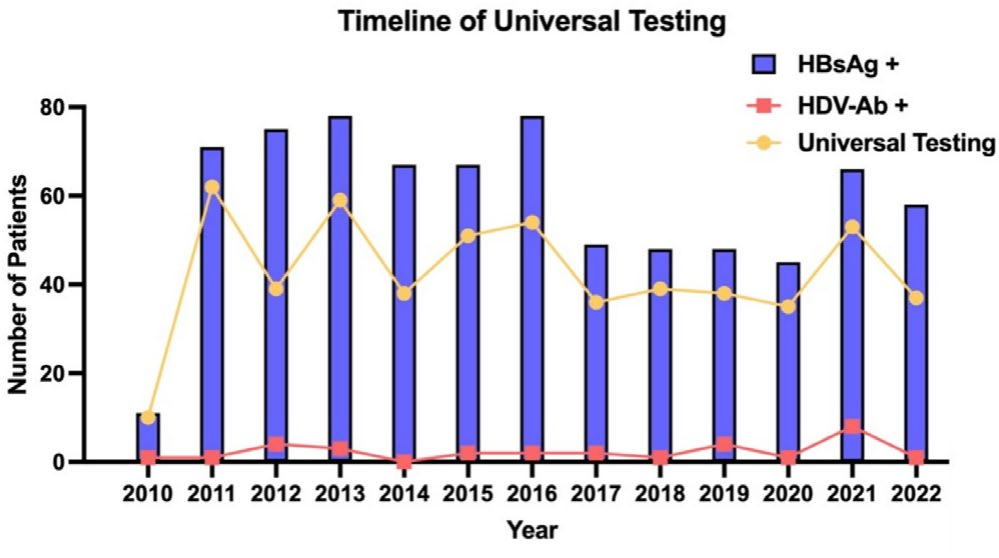
Implementation of the universal screening policy at Soroka University Medical Center and anti‐HDV detection between 2010 and 2022. HDV, hepatitis D virus.

**FIGURE 2:**
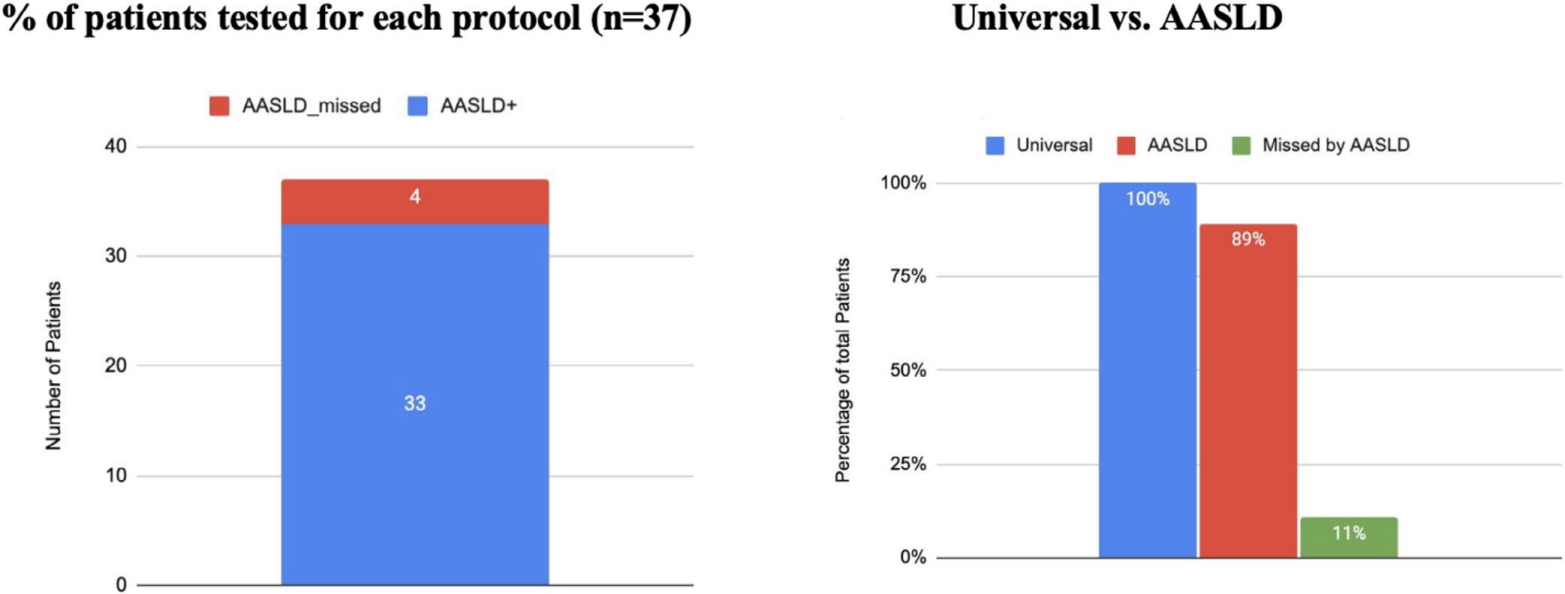
Percentage of anti‐HDV‐positive patients detected by universal screening in comparison to AASLD risk‐based screening. AASLD, American Association for the Study of Liver Diseases; HDV, hepatitis delta virus.

During the study period between January 2010 to September 2022, the SUMC virology lab identified 761 patients who tested positive for HBsAg (Figure S1). There were no exclusions due to missing data. The majority of patients were male (428, 56%, Table 1). Mean age during diagnosis was 57 years (42–71). 547 patients (73%) were Jewish, 202 (27%) were Arab and 12 (1.5%) were of unknown ethnicity. 51 patients (6.7%) were found positive for IgM HBCore. 525 (69%) patients met the AASLD criteria for HDV screening (AASLD high‐risk group, AHR) and 236 (31%) did not (AASLD low‐risk group, ALR). Universal screening was performed on 559 (73.4%) patients (Figure 1). The median time of testing for anti‐HDV following HBsAg detection was 0 days (Figure S2). It identified 37 patients positive for anti‐HDV (6.6%). None of them were positive for IgM HBCore. 17 patients (45.9%) had quantifiable or qualitative proof of HDV RNA positivity. In the AHR group, HDV antibody positivity was found in 33 patients (8.6%). 17 (51.5%) were found to be HDV RNA‐positive. In the ALR group, 4 (2.3%) were found to be anti‐HDV‐positive. None were found to be HDV RNA‐positive with a qualitative or quantifiable test. Thus, screening based on the AASLD criteria provided a sensitivity of only 89% for HDV antibody‐positive patients (Figure 2). AASLD risk‐based screening was able to identify all 17 (4.4%) HDV RNA‐positive patients. The specificity, positive predictive value and negative predictive value of AASLD based screening in detecting anti‐HDV‐positive patients was 33.1%, 8.6% and 97.7% respectively.

**TABLE 1:** Patient characteristics.

	Total (*n* = 761)^a^	AASLD low‐risk group (*n* = 236)^a^	AASLD high‐risk group (*n* = 525)^a^	*p* ^b^
Male	428 (56%)	111 (47%)	317 (60%)	< 0.001
Ethnicity				
Arab	202 (27%)	147 (62%)	55 (11%)	< 0.001
Jewish	547 (73%)	89 (38%)	458 (89%)	
Unknown	12	0	12	
Age	57 (42, 71)	47 (36, 61)	62 (44, 74)	< 0.001
History of alcohol use	33 (5.2%)	6 (3.0%)	27 (6.1%)	0.10
Active smoking	179 (28%)	42 (21%)	137 (31%)	0.009
Elevated ALT^c^	153 (20%)	33 (14%)	120 (23%)	0.005
Elevated AST^c^	305 (41%)	79 (34%)	226 (44%)	0.014
FIB‐4	1.54 (0.89, 3.11)	1.05 (0.71, 2.58)	1.70 (1.08, 3.25)	< 0.001
Positive HBeAg	97 (13%)	34 (15%)	63 (12%)	0.3
IgM positive HBCore	51 (6.7%)	24 (10.1%)	27 (6.9%)	0.011
HBV DNA (IU/mL)	487 (108, 5824)	1007 (206, 8816)	372 (90, 3769)	0.023
NAs treatment				
Entecavir	104 (14%)	32 (14%)	72 (14%)	0.5
Lamivudine	101 (13%)	33 (14%)	68 (13%)	
Tenofovir DF	85 (11%)	32 (14%)	53 (10%)	
No treatment	471 (62%)	139 (59%)	332 (63%)	
All‐cause mortality	239 (31%)	48 (20%)	191 (36%)	< 0.001

^a^ *n*(%); Median (IQR).

^b^ Pearson's Chi‐squared test; Wilcoxon rank sum test.

^c^ Above two times upper limit of norm.

### 3.2 High‐Risk AASLD Criteria Group

In the AHR group (525 patients, Table 1), most patients were male (317, 60%), and the vast majority were of Jewish ethnicity (89%). Mean age was 62 years (44–74). The majority of patients were HBeAg‐negative (451, 86%). Elevated levels of ALT and AST were found in 23% and 44% of patients, respectively. The mean FIB‐4 score was 1.70 (1.08, 3.25). Mean HBV DNA was 372 (90, 3769) IU/mL. 16 patients (3.1%) fulfilled the HIV co‐infection criteria (Table 2). 100 patients (19%) were found to be co‐infected with HCV. 18 patients (3.4%) had a history of drug injection. 22 patients (4.2%) had any history of sexually transmitted disease. 433 patients (82%) were born in regions with reported high HDV endemicity. Most patients (202, 38%) in the AHR group originated from states once affiliated with the former Soviet Union (Table S1). 97 (40%) patients fulfilled the criteria of elevated ALT or AST and low or undetectable HBV DNA. Data regarding sexual practices were unavailable from the HMO medical files. 191 (36%) patients from the AHR group expired during the study period from any cause.

**TABLE 2:** Fulfilment of the AASLD high‐risk criteria for HDV screening.

	Total (*n* = 761)^a^	AASLD low‐risk group (*n* = 236)^a^	AASLD high‐risk group (*n* = 525)^a^
Region of birth			
Africa	75 (9.9%)	0 (0%)	75 (14%)
Asia	22 (2.9%)	0 (0%)	22 (4.2%)
Former USSR	202 (27%)	0 (0%)	202 (38%)
Israel and Europe	299 (39%)	232 (98%)	67 (13%)
Middle East	134 (18%)	0 (0%)	134 (26%)
Caribbean	4 (0.5%)	2 (0.8%)	2 (0.4%)
Other	25 (3.3%)	2 (0.8%)	23 (4.4%)
IV drug use	18 (2.8%)	0 (0%)	18 (4.1%)
Infection with HCV	100 (13%)	0 (0%)	100 (19%)
Infection with HIV	16 (2.1%)	0 (0%)	16 (3.1%)
History of STD	22 (2.9%)	0 (0%)	22 (4.2%)
Elevated ALT/AST with low or undetectable HBV DNA	97 (12.7%)	0 (0%)	97 (18.5%)

Abbreviations: ALT, alanine aminotransferase; AST, aspartate aminotransferase; HCV, hepatitis C virus; HIV, human immunodeficiency virus; STD, sexually transmitted disease.

### 3.3 Low‐Risk AASLD Criteria Group

**FIGURE 3:**
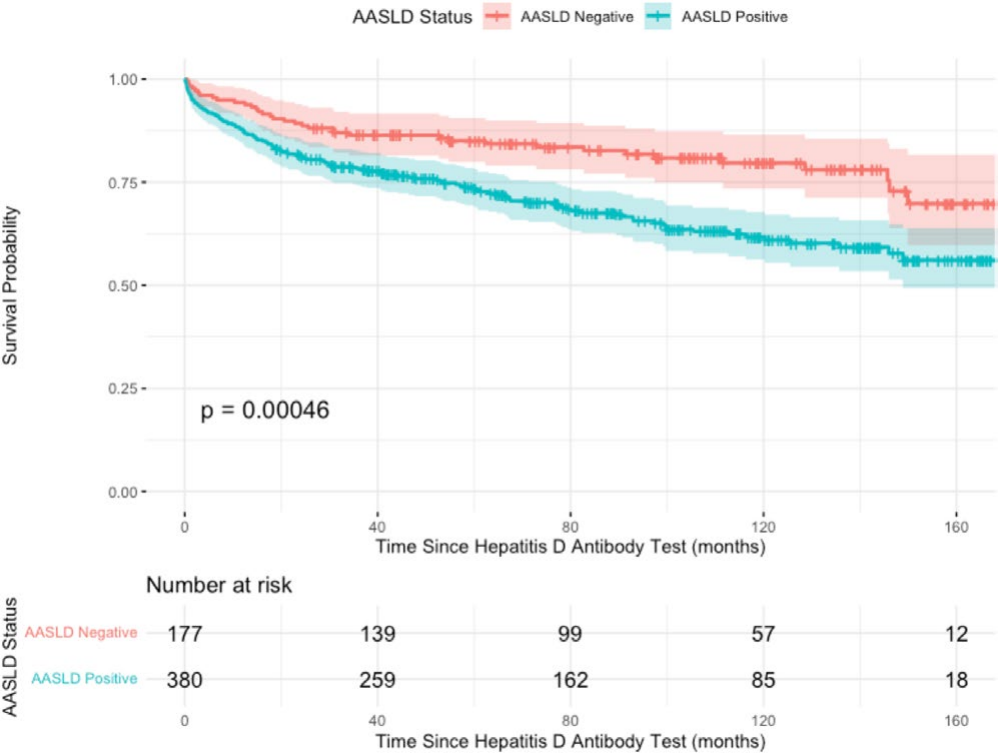
Kaplan–Meier curve of overall survival duration estimates by AASLD low‐risk and high‐risk groups during the study period. Median follow‐up time was 26 months.

In comparison with the AHR group, in the ALR group (236 patients), most patients were female (53%, *p* < 0.001), and the majority were of Arab ethnicity (62.3%, *p* < 0.001). Mean age was significantly lower than that of the AHR group, 47 years (36–61, *p* < 0.001). Smoking was significantly less common (21% vs. 31%, *p* = 0.009). Both ALT and AST were significantly less elevated in the ALR group in comparison to the AHR group. In addition, the mean FIB‐4 score was significantly lower in the ALR group in comparison to the AHR group (1.05 (0.71, 2.58) vs. 1.70 (1.08, 3.25), *p* < 0.001). There were no differences in HBeAg positivity (15% vs. 12%, *p* = 0.3). There were no differences in NA treatment upon the initial HBV diagnosis (*p* = 0.5). 48 (20%) patients from the ALR group expired during the study period from any cause. This mortality rate was found to be statistically significantly less than that in the AHR group (*p* < 0.001). A Kaplan–Meier survival analysis revealed a significant association between the AASLD risk status and mortality (Figure 3). In a multivariable analysis, AHR status exhibited a hazard ratio of 1.92 (1.32–2.78, *p* < 0.001), indicating a 92% higher risk of all‐cause mortality compared to the ALR group.

### 3.4 Severity of Liver Disease

Despite significantly more patients found with elevated ALT and AST in the AHR group in comparison to the ALR group, a comparison between the groups found no major differences regarding clinical deterioration attributed to advanced liver disease. There were no statistically significant differences (Table 3) between the groups in their presentation with cirrhosis (*p* = 0.3), jaundice (*p* = 0.4), ascites (*p* = 0.3) or encephalopathy (*p* = 0.4). A comparison of major liver outcomes (MALO) during the entire study period also revealed no statistically significant differences in additional outcomes such as the presence of bleeding or non‐bleeding oesophageal varices, hepatorenal syndrome, a diagnosis of HCC, liver transplantation and liver‐related death. In addition, a multivariable analysis found that age of patients (HR 1.07,1.06–1.08, *p* < 0.001), active smoking (HR 1.65, 1.12–2.41, *p* = 0.01) and initiation of antiviral medications upon HBV infection diagnosis (HR 1.96, 1.33–2.88, *p* < 0.001) all increase the HR for all‐cause mortality. Obesity was found to have a non‐statistically significant trend towards increased all‐cause mortality (HR 1.42, 0.96–2.11, *p* = 0.07). Alcohol use, socioeconomic status and the presence of diabetes were found not to have any significant impact on all‐cause mortality (*p* = 0.4, *p* = 0.6, *p* = 0.7, respectively).

**TABLE 3:** Comparison of liver disease severity and major liver outcomes between high‐risk and low‐risk AASLD groups during initial presentation and throughout the study period.

	Total (*n* = 761)^a^	AASLD low risk group (*n* = 236)^a^	AASLD high risk group (*n* = 525)^a^	*p* ^b^
During initial presentation				
Cirrhosis	38 (5%)	7 (3%)	31 (5.9%)	0.3
Jaundice	69 (9.1%)	24 (10%)	45 (8.6%)	0.4
Ascites	38 (5%)	7 (3%)	31 (5.9%)	0.3
Encephalopathy	9 (1.2%)	3 (1.2%)	6 (1.1%)	0.4
Major liver outcomes (MALO) during the study period				
Cirrhosis	69 (9.1%)	18 (7.8%)	51 (9.7%)	0.4
Oesophageal varices (not bleeding)	24 (3.2%)	9 (3.9%)	15 (2.9%)	0.5
Encephalopathy	20 (2.6%)	10 (4.3%)	10 (1.9%)	0.056
Ascites	55 (7.3%)	13 (5.6%)	42 (8.0%)	0.2
Hepatorenal syndrome	5 (0.7%)	0 (0%)	5 (1%)	0.3
Oesophageal varices (bleeding)	15 (2%)	5 (2.2%)	10 (1.9%)	0.8
Hepatocellular carcinoma	22 (2.9%)	9 (3.8%)	13 (2.5%)	0.3
Liver transplantation	3 (0.4%)	2 (0.9%)	1 (0.2%)	0.2
Liver‐related death	41 (5.4%)	12 (5.1%)	29 (5.5%)	0.87

^a^ *n*(%).

^b^ Pearson's Chi‐squared test.

## 4 Discussion

In this retrospective study, we evaluated the impact of universal screening on HDV detection in a single tertiary medical center in comparison to the more conservative risk‐based screening endorsed by the 2018 AASLD guidelines regarding the diagnosis of HDV in chronic HBV patients. The results of our study support the utilisation of universal screening for HDV in all HBV patients, as risk‐based screening identified 11% fewer HDV antibody‐positive patients than the number of patients identified while utilising universal screening. In our study, risk‐based screening led to an HDV detection rate of 89%. This was achieved after careful review of patients' medical records in search of known risk factors for HDV infection. However, in real life, low awareness of physicians to one or more of the risk factors for HDV infection, especially in low prevalence regions, may lead to lower HDV screening practices with subsequent higher miss rates of HDV detection. In addition to our main findings, we were also able to demonstrate that patients stratified as high risk via the AASLD criteria presented with significantly higher ALT and AST levels, representing a more severe hepatitis, as well as a higher mean FIB‐4 score suggesting advanced hepatic fibrosis. In addition, in a multivariable analysis, they were found to have an increased risk for all‐cause mortality. Finally, patients screened for anti‐HDV in the AHR group were more likely to have detectable HDV RNA viral load by PCR.

The AASLD guidance statements for the management of patients with chronic HBV infection by Terrault et al. [17] were originally published in 2018 and presented an HDV screening algorithm designed for a relatively rare disease in the western hemisphere, an infection that most clinicians will probably not encounter unless treating a high risk population such as intravenous drug users or while caring for patients originating from countries with an increased prevalence of the infection [22, 23]. This conservative approach made sense in the context it was written. Most studies evaluating the global prevalence of HDV estimated only 15–20 million people were infected with the virus worldwide [24]. However, later studies published after 2017 significantly raised the estimated prevalence of HDV infection to more than 70 million persons worldwide [11, 25]. Not surprisingly, in the more recently published EASL clinical practice guidelines on HDV from 2023 authors recommended screening for anti‐HDV in all HBsAg‐positive patients at least once [18]. This decision by the authors was based on the growing evidence accumulating from recent publications regarding the ‘tip of the iceberg’ phenomenon in HDV infection [26, 27], supporting the more proactive universal screening approach in HDV detection even in populations considered to be low risk for HDV prevalence such as the United States [28].

In our study, we were able to very clearly demonstrate the benefit of universal screening, which began its implementation in SUMC starting from 2010. A previous study from Israel found a significant 6.5% prevalence of HDV among HBsAg‐positive patients [29] which prompted a yet to be fulfilled call for a national universal screening policy. This prevalence is similar to the one reported in France, where the introduction of reflex testing for anti‐HDV significantly increased the detection rate of HDV among new HBsAg‐positive patients [30]. However, our study revealed that even in our own tertiary center, compliance with the universal screening protocol was not always upheld (Figure 3) as only about 73% of new HBsAg‐positive patients in our cohort were screened. The reason for this disparity is most likely due to laboratory protocol limitations, which required a separate blood draw after the initial HBsAg positivity. However, a lack of adherence to the screening policy cannot be completely excluded. Whatever the reason for the incomplete implementation of the universal screening policy at SUMC was, its importance in identifying HDV patients is clear, especially as therapeutic options become available [3, 4] for this once untreatable disease. Interestingly, our study was also able to show that patients identified by the AASLD high risk criteria were more likely to harbour a more severe hepatitis and, even worse, were more likely to succumb to mortality from any cause. These findings could perhaps show the utility of the AASLD criteria in identifying patients at risk for a more severe disease and a worse outcome rather than as a general screening tool in chronic HBV patients.

The main strength of our study stems from the relatively large database of HBV patients who were diagnosed for the first time with a positive HBsAg and then tested for HDV in the SUMC virology lab. In addition, the electronic medical record (EMR) for each patient's evaluation at SUMC is connected to the CHS primary care clinics data sources and therefore allowed for a robust data collection regarding the various AASLD risk factors evaluated. This connection also allowed us to obtain the long‐term clinical outcomes of patients evaluated as well as the severity of liver disease during the initial presentation and during the long‐term follow‐up.

The main limitation of our study is its retrospective design and therefore the lack of adherence to the universal screening policy by our lab. During the initial conception of this study, we estimated that compliance with universal screening for anti‐HDV by the SUMC virology lab to be well above 90%. Surprisingly, only approximately 73% of new HBsAg‐positive patients diagnosed at the SUMC lab were actually tested for HDV antibodies. This finding placed a limitation on our goal to fully compare the impact of universal screening on newly diagnosed HBV patients in comparison to the traditional risk‐based screening advocated by the 2018 AASLD guidelines. However, despite these significant limitations, we were able to show a significant 11% increase in anti‐HDV detection in HBV patients in comparison to risk‐based screening. In addition, we were unable to completely utilise the AASLD criteria on our study cohort due to lack of data regarding sexual practices in the EMR. Another important limitation of our study is our inability to demonstrate the cost–benefit advantage of universal screening. Since HDV infection has no currently approved therapy in Israel, we were unable to demonstrate the clinical significance of the utilisation of universal screening in our cohort of patients. However, since several therapeutic trials are currently in different stages of development, it is our opinion that early diagnosis of HDV infection could offer the identified patients a chance to participate in such clinical trials and alter the course of their disease. Finally, our observations stem from a single tertiary center in Israel with a diverse population of Jews and Muslim Bedouins. The virology lab of SUMC serves as a reference lab for HDV testing for the entire Southern region of Israel. Therefore, the results of this study are actually representative of approximately 15% of the population of Israel. Notwithstanding, unique demographic and healthcare infrastructure features of this region may indeed differ from other parts of Israel, limiting the generalisability of our findings.

In conclusion, in this retrospective cohort study we have demonstrated the performance of universal screening is superior to the 2018 AASLD risk‐based screening recommendations for the detection of HDV infection among HBV patients. Our findings advocate for the widespread acceptance of universal screening among HBV patients as the preferred method of screening to allow timely identification of patients at risk for a more severe disease. We believe better detection of HDV patients will allow for earlier access to novel treatment options and better surveillance of hepatic complications.

## Author Contributions

All authors contributed to the writing and editing of the manuscript and approve the final version. D.Y.: Conception, writing of manuscript, approval of final draft. O.C.: Formal analysis, resources, writing – review and editing. B.I.: Formal analysis, resources, writing – review and editing. I.L.: Resources, writing – review and editing. A.A.J.: Resources, writing – review and editing. N.A.: Resources, writing – review and editing. A.K.N.: Resources, writing – review and editing. N.E.: Resources, writing – review and editing. A.N.S.: Resources, writing – review and editing. O.E.: Conception, writing of manuscript, senior supervision, approval of final draft.

## Conflicts of Interest

The authors declare no conflicts of interest.

## Supporting information


**Figure S1.** Study population flow chart. HBsAg, Hepatitis B surface antigen; AASLD, American association for the study of liver diseases; HDV‐Ab, Hepatitis delta virus antibodies.

**Figure S2.** HDV antibody testing frequency following HBsAg detection. HDV, Hepatitis delta virus; HBsAg, Hepatitis B surface antigen.

**Table S1.** Region of birth and HDV endemicity for all patients evaluated in the study. HDV, Hepatitis delta virus.

## Data Availability

The data that support the findings of this study are available from the corresponding author upon reasonable request.
