# Supplementary figures and images for: Impact of Universal Screening for HDV in HBV‐Infected Patients on Chronic HDV Detection Rate in Israel
Source: J Viral Hepat. 2025 Jun 11;32(7):e70046. doi: 10.1111/jvh.70046 (PMC12153417; doi:10.1111/jvh.70046)

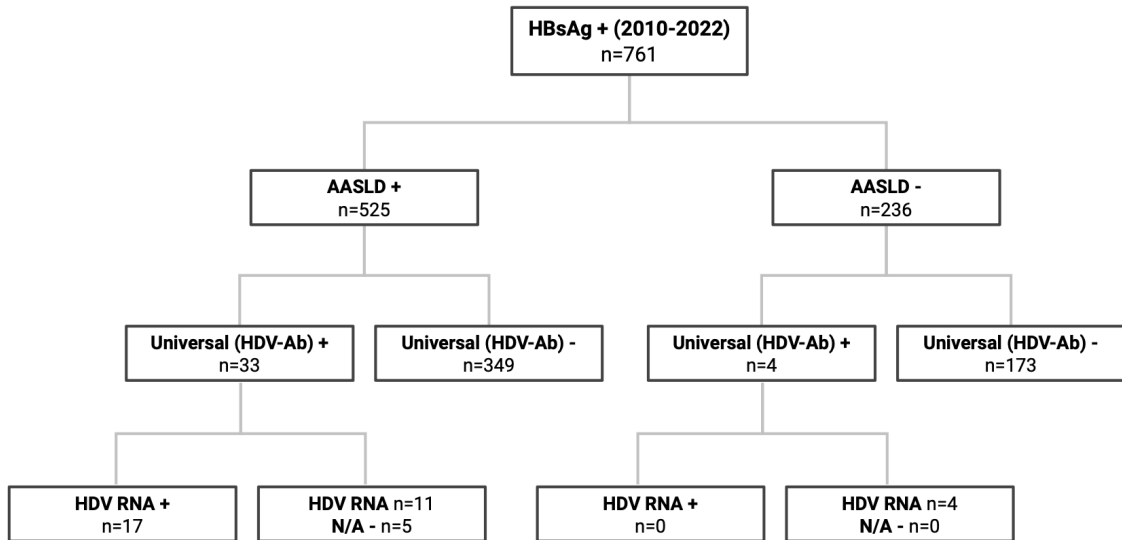

Supplement: Supplementary file 1 — Figure S1. Study population flow chart. HBsAg, Hepatitis B surface antigen; AASLD, American association for the study of liver diseases; HDV‐Ab, Hepatitis delta virus antibodies. [file JVH-32-0-s003.pdf]

# Time to Reflex Testing

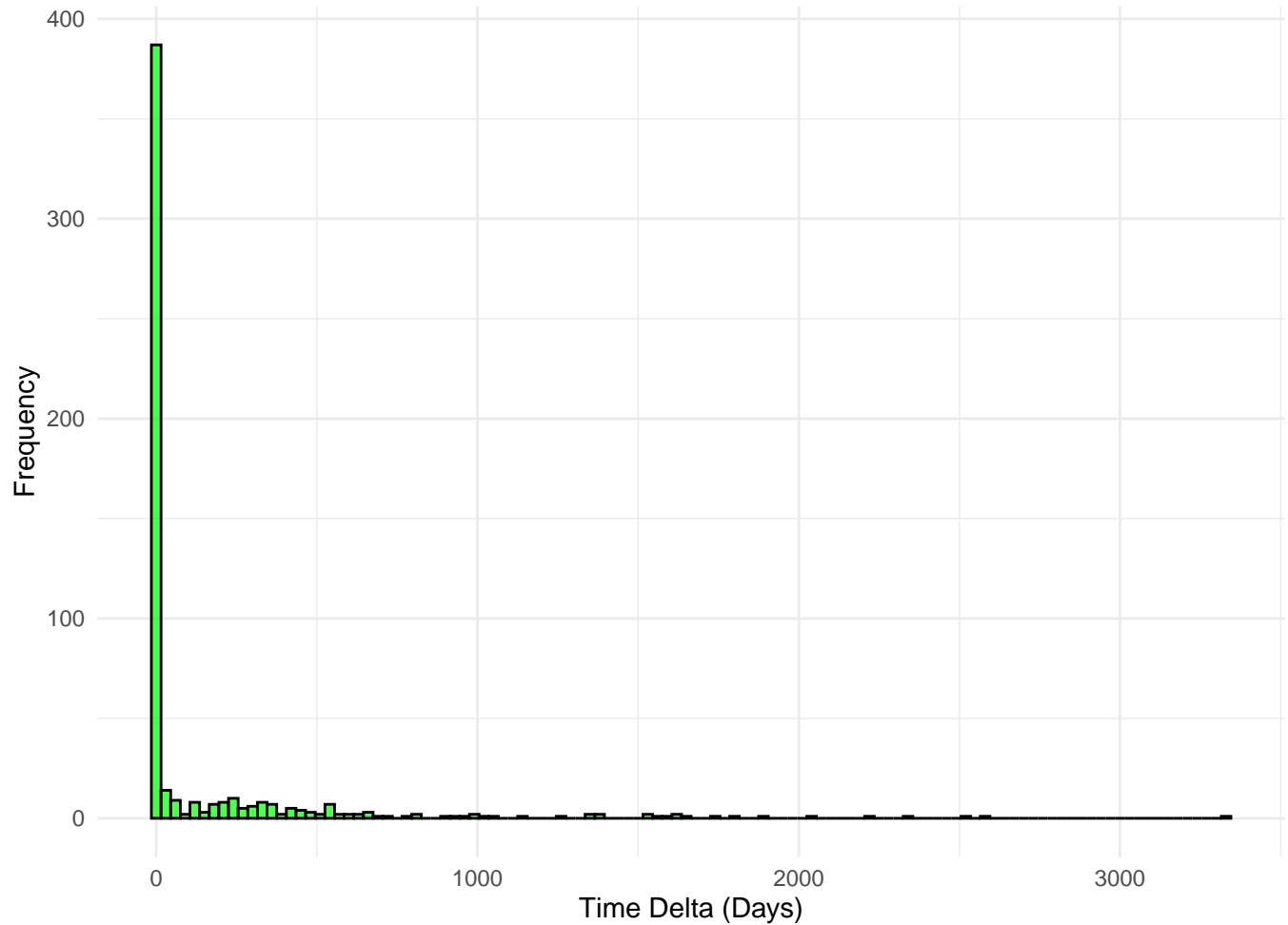

Supplement: Supplementary file 2 — Figure S2. HDV antibody testing frequency following HBsAg detection. HDV, Hepatitis delta virus; HBsAg, Hepatitis B surface antigen. [file JVH-32-0-s002.pdf]
